# Evaluation of semiempirical VMAT dose reconstruction on a patient dataset based on biplanar diode array measurements

**DOI:** 10.1120/jacmp.v15i2.4705

**Published:** 2014-03-06

**Authors:** Cassandra Stambaugh, Daniel Opp, Stuart Wassrman, Geoffrey Zhang, Vladimir Feygelman

**Affiliations:** ^1^ Department of Physics University of South Florida Tampa FL; ^2^ Department of Radiation Oncology Moffitt Cancer Center Tampa FL USA

**Keywords:** VMAT QA, diode array, measurement‐guided dose reconstruction, patient dose reconstruction

## Abstract

We report the results of a preclinical evaluation of recently introduced commercial tools for 3D patient IMRT/VMAT dose reconstruction, the Delta^4^ Anatomy calculation algorithm. Based on the same initial measurement, volumetric dose can be reconstructed in two ways. Three‐dimensional dose on the Delta^4^ phantom can be obtained by renormalizing the planned dose distribution by the measurement values (D4 Interpolation). Alternatively, incident fluence can be approximated from the phantom measurement and used for volumetric dose calculation on an arbitrary (patient) dataset with a pencil beam algorithm (Delta^4^ PB). The primary basis for comparison was 3D dose obtained by previously validated measurement‐guided planned dose perturbation method (ACPDP), based on the ArcCHECK dosimeter with 3DVH software. For five clinical VMAT plans, D4 Interpolation agreed well with ACPDP on a homogeneous cylindrical phantom according to gamma analysis with local dose‐error normalization. The average agreement rates were 98.2%±1.3% (1 SD), (range 97.0%‐100%) and 92.8%±3.9% (89.5%‐99.2%), for the 3%/3 mm and 2%/2 mm criteria, respectively. On a similar geometric phantom, D4 PB demonstrated substantially lower agreement rates with ACPDP: 88.6%±6.8% (81.2%‐96.1%) and 72.4%±8.4% (62.1%‐81.1%), for 3%/3 mm and 2%/2 mm, respectively. The average agreement rates on the heterogeneous patients' CT datasets are lower yet: 81.2%±8.6% (70.4%‐90.4%) and 64.6%±8.4% (56.5%‐74.7%), respectively, for the same two criteria sets. For both threshold combinations, matched analysis of variance (ANOVA) multiple comparisons showed statistically significant differences in mean agreement rates (p<0.05) for D4 Interpolation versus ACPDP on one hand, and D4 PB versus ACPDP on either cylindrical or patient dataset on the other hand. Based on the favorable D4 Interpolation results for VMAT plans, the resolution of the reconstruction method rather than hardware design is likely to be responsible for D4 PB limitations.

PACS number: 87.55Qr

## I. INTRODUCTION

It is a current standard of practice to perform a patient‐specific, end‐to‐end test for each intensity‐modulated radiation therapy (IMRT), or volumetric‐modulated arc therapy (VMAT) plan.^(1)^ The agreement between the measured and planned dose distributions is typically quantified by some combination of the percent dose error and distance‐to‐agreement (DTA) criteria, most often in the form of gamma index (γ)‐analysis.^(2)^ While reliable agreement between the calculated and measured/reconstructed dose in a geometrical phantom is the basis for the dosimetric commissioning of an IMRT system, its value for the meaningful patient‐specific, end‐to‐end testing is less clear. With the prevalent 3% globally normalized dose error and 3 mm DTA threshold criteria in particular,^3^, ^4^, ^5^ y‐analysis passing rates for either per‐beam, single‐plane^(6)^ or quasi‐3D^(7)^ array geometries, had weak — and counter‐intuitive, if any — correlation with the conventional clinical DVH metrics. Instances of the 3%/3 mm passing rate metric's failure to detect systematic errors are numerous.^6^, ^7^, ^8^, ^9^ On the other hand, direct comparison of the planned and deliverable DVHs exhibits higher sensitivity and specificity, and is expected to be more clinically meaningful and intuitive to both the physician and the physicist.^7^, ^10^, ^11^ Semiempirical volumetric dose reconstruction, based on the array measurements, was previously reported on geometrical phantoms^12^, ^13^, ^14^, ^15^ and patient CT datasets.^16^, ^17^ Following this trend, a recently released Delta^4^ (ScandiDos AB, Uppsala, Sweden) software module called Anatomy was purchased at our clinic. It allows semiempirical IMRT/VMAT dose reconstruction on the patient CT, based on the phantom measurements from the Delta^4^ biplanar diode array dosimeter. In this paper, the results of the initial tests of the system, focusing on its performance in VMAT dose reconstruction are presented.

## II. MATERIALS AND METHODS

### A. General treatment planning and delivery

Dose calculations were performed with Pinnacle treatment planning system (TPS) v 9.2 (Philips Radiation Oncology Systems, Fitchburg, WI) using collapsed cone convolution algorithm. The test plans were arranged in an order of increasing complexity, starting with profile comparisons for static rectangular and bar‐pattern^(18)^ fields and progressing to VMAT dose comparisons, first on a homogeneous cylindrical phantom and then on a patient CT dataset. For the VMAT tests, five plans previously treated at our institution were selected: three head and necks of varying complexity (single PTV versus two targets with simultaneous integrated boost) with conventional fractionation (2 Gy/fraction), one pancreas stereotactic body radiation therapy (SBRT) plan (6 Gy/fraction), and one lung SBRT (10 Gy/fraction). All VMAT plans were calculated on a 2.5 mm dose grid with 4° control point (CP) angular increment.^(19)^ All plans employed a 6 MV beam from a TrueBeam linear accelerator equipped with a 120 leaf Millennium MLC (Varian Medical Systems, Palo Alto, CA).

### B. Volumetric dose reconstruction methods

#### B.1 Delta^4^ dose reconstruction

##### B.1.1 Direct measurement at the diodes' locations

This basic functionality of the Delta^4^ system is by now well described and validated.^14^, ^20^, ^21^ The dosimeter has two planar diode arrays arranged at a right angle and thus the 2D beam modulation information is preserved regardless of the gantry angle. The detector spacing varies from 5 mm in the center of the $20\times 20\;{\text{cm}}^{2}$ active area to 10 mm on the periphery. In its basic implementation, the measured dose at the detector positions is compared to the planned dose (on the Delta^4^ cylindrical phantom) extracted from the DICOM RT DOSE object transmitted from the TPS.

##### B.1.2 Volumetric interpolation inside the Delta^4^ phantom

The next step towards a more comprehensive evaluation is 3D dose reconstruction on the native Delta^4^ phantom, which is a 22 cm diameter cylinder, most often made from Polymethyl metacrylate (PMMA). This volumetric dose reconstruction is fairly straightforward and has been previously validated.^14^, ^15^, ^21^ For each plan CP, the rays are traced through the measurement points, and the TPS dose calculated on the Delta^4^ phantom along each ray is renormalized to fit the measurement. No additional beam characterization is necessary if the TPS dose data at the CP level is available. This method of volumetric dose reconstruction on the Delta^4^ phantom will be referred to as “D4 Interpolation”.

##### B.1.3 Dose reconstruction on an external patient CT dataset

This is yet another step up in complexity, and accuracy of this method is the main subject of the current investigation. The detailed description of the algorithm is available in the recently released vendor's White Paper.^(22)^ Patient dose reconstruction is a two‐step process. First, the most likely fluence that would result in the measured Delta^4^ dose distribution is estimated through optimization. Then, the obtained energy fluence per control point (CP) is used as an input parameter to calculate volumetric dose on the patient CT dataset with a pencil beam (PB) algorithm.^(23)^ The energy fluence estimation is formulated as a linear programming problem: find the minimum area integral of energy fluence given that the calculated dose in the Delta^4^ phantom is larger than, or equal to, the measured dose in all measurement points. The energy fluence matrix pixel size is $6\times 6\;{\text{mm}}^{2}$.^(22)^ Dose at a point in the patient is computed by integrating the energy fluence with the primary, scatter, and charged particle contamination kernels in a 2D plane containing the calculation point that is perpendicular to the central axis of the beam.

To perform this calculation, the beam must be first characterized in the Delta^4^ software. Specifically, percent depth doses on a water phantom and in‐air relative output factors $\left(S_{c}\right)$ are required for a set of field sizes, typically from 2×2 to $20\times 20\;{\text{cm}}^{2}$. The patient CT, dose, and structures are imported from the TPS as DICOM RT objects. ACT number to chemical composition assignment scheme, typically reserved for more sophisticated algorithms such as Monte Carlo,^(24)^ is used to estimate radiological depth for PB calculations. No option to override CT densities is provided. Once the calculation is complete, the patient reconstructed 3D dose grid can be compared to the planned one with a set of standard analysis tools, such as dose profiles, 3D γ‐analysis, and dose‐volume histogram (DVH) comparisons for selected structures. This technique will be referred to as the dose reconstruction method “D4 PB”.

#### B.2 3DVH dose reconstruction

The primary comparison method used to volumetrically evaluate accuracy of the D4 PB reconstruction algorithm is measurement‐guided dose reconstruction by 3DVH software (v. 3.0) based on the ArcCHECK diode array (Sun Nuclear Corp., Melbourne, FL) measurements. This method, called ArcCHECK planned dose perturbation (ACPDP), was described in detail and evaluated previously on geometrical and anthropomorphic phantoms using dose sampling with ion chambers, film, and optically stimulated luminescence dosimeters.^17^, ^25^, ^26^ Recently, a full 3D dose ACPDP validation with BANG3 polymer gel (MGS Research, Guilford, CT) was also reported.^(27)^

The ArcCHECK has 1386 diodes arranged in a helical pattern and, in the beam's eye view (BEV), the detector configuration is essentially invariant with the gantry angle.^(25)^ Dose acquisition is controlled by SNC Patient software (v. 6.2, Sun Nuclear). For VMAT dose recon‐struction, ACPDP explicitly relies on the time‐resolved nature of the ArcCHECK data, with updates logged at 50 ms intervals.^(17)^ Along with the dose, the gantry angle determined by the virtual inclinometer^(25)^ is also stored for each data update. At this point, both the low resolution (~10 mm spacing) dose map on the cylindrical ArcCHECK surface and the gantry angle are known as a function of time. The DICOM RT Plan's beams' CPs are thus synchronized to absolute, corresponding delivery times, forming the basis of discretizing the delivery process into individual modulated subbeams at $\sim 2^{{}^{\circ}}$ intervals (which typically corresponds to resolution of 0.2−0.4 sec in terms of delivery time).

A relative 3D dose grid for each subbeam is independently calculated by convolving a 3D impulse TERMA function throughout the phantom volume with the 3D scatter depth kernels.

The next step is the position‐dependent, measurement‐guided dose morphing and absolute scaling that converts full‐density relative dose to absolute, using the relevant diode measurements as 3D spatial calibration data points. After that, a full‐volume, high‐resolution absolute dose grid is generated on the ArcCHECK phantom by summing all the component time‐resolved subbeam dose grids. The final step is to use this high‐density volumetric phantom dose grid to obtain measurement‐driven estimate of the dose delivered to the patient. To that end, the voxel‐by‐voxel correction factors derived from the ratios of calculated (TPS) and reconstructed doses on the phantom are applied to the TPS dose distribution on the patient CT.

### C. Specific tests

While the primary goal was to compare D4 PB to ACPDP, the D4 Interpolation and TPS data were also collected and analyzed when appropriate.

#### C.1 Static fields

Two static MLC‐defined field arrangements from our standard commissioning set were used: a $2\times 2\;{\text{cm}}^{2}$ square and a bar pattern (a set of 2 cm wide openings separated by 2 cm areas of closed leaves).^(18)^ In each case, a scan at 100 cm source‐to‐surface distance and 10 cm depth was obtained in a water tank with a Model PFD‐3G diode (IBA Dosimetry GmbH, Schwarzenbruck, Germany). The diode's sensitive volume diameter is 2 mm. The bar pattern was scanned in the Y direction (in‐plane), while the square field was scanned in the X direction (cross‐plane), corresponding to the MLC leaf movement direction. For the latter, the scan position was offset from the central axis by 2.5 mm to scan in the middle of the leaf. The same plans were delivered in a standard fashion^19^, ^25^ to the calibrated Delta^4^ and ArcCHECK dosimeters. D4 PB and ACPDP reconstructions were performed on a CT scan of the rectangular Plastic Water (CIRS Inc., Norfolk, VA) phantom (“patient”). The diode scans were compared with the relative dose profiles extracted from the D4 PB and ACPDP reconstructions, and TPS calculations.

In addition, the D4 Interpolation and ACPDP reconstructions were performed on a PMMA cylinder. The profiles from those data and the TPS‐calculated profiles were compared at the 100 cm source‐to‐axis distance. All calculations and reconstructions were done on a 2 mm dose grid.

#### C.2 VMAT dose reconstruction on homogeneous phantoms

##### C.2.1 PMMA Delta^4^ cylindrical phantom

**Figure 1:**
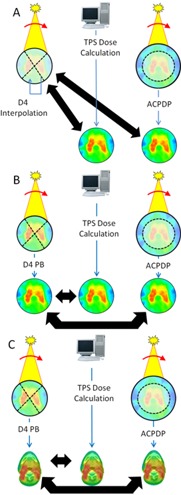
Delta^4^ dose (a) is either directly measured (at the detectors) or interpolated (D4 Interpolation) inside the native PMMA phantom. The ACPDP (reconstructed) and TPS (calculated) dose distributions compared to the Delta^4^ are also on the same PMMA cylinder. Delta^4^ Pencil Beam reconstruction (b) (D4 PB), ACPDP reconstruction, and TPS calculation are compared on the water‐equivalent cylindrical phantom; (c) same as for (b), but comparisons are done on the corresponding patients' CT datasets.

The phantom density value relative to water was set to 1.147 in the TPS and the dose was calculated. Delta^4^ and ArcCHECK VMAT measurements were performed. For each plan, ACPDP reconstruction was performed on a cylindrical PMMA phantom (“patient”). First, the samples of the ACPDP and TPS 3D dose grids were compared to the directly measured Delta^4^ dose at the diode's locations. Then the ACPDP and TPS volumetric doses were compared to the D4 Interpolation 3D dose grid (Fig. 1(a)). All comparisons here and elsewhere in the manuscript used γ‐analysis of absolute dose distributions with local (at the evaluated point) dose error normalization. Passing rates with both 3%/3 mm and 2%/2 mm threshold combinations are reported. Dose points receiving less than 10% of the maximum dose were excluded from evaluation.^ All γ‐analyses were performed in 3D, as implemented in the Delta^4^ software (February 2013 release). This was the only readily available option since there is no DICOM RT DOSE object export capability with Delta^4^. Representative dose profiles were exported to illustrate crucial areas of disagreement.

##### C.2.2 Water‐equivalent Delta^4^ cylindrical virtual phantom

Since the D4 PB algorithm uses a complicated CT to density conversion scheme,^(22)^ we decided to do the comparisons of D4 PB with ACPDP on a unit density cylindrical phantom, to eliminate the additional uncertainty associated with the CT number to chemical composition assignment. The virtual Delta^4^ phantom supplied by the manufacturer was modified programmatically so that the Hounsfield units were uniformly set to zero. This phantom was used as “patient” for D4 PB and ACPDP reconstructions, and also for the TPS calculation (Fig. 1(b)). The resulting volumetric dose grids were compared three ways, as described above.

##### C.3 VMAT dose reconstruction on patient CT datasets

The same dose reconstruction and comparison procedure were used, except an appropriate patient CT dataset was substituted for the Delta^4^ phantom for each case (Fig. 1(c)).

## III. RESULTS

### A. Static fields

**Figure 2:**
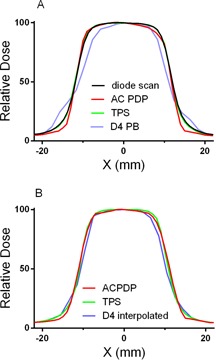
Relative lateral dose profiles in X direction (cross‐plane) for a $2\times 2\;{\text{cm}}^{2}$ MLC‐defined field. (a): SSD 100 cm, depth 10 cm. The diode scan was taken in water, ACPDP and D4 PB reconstruction and TPS calculation are all on a Plastic Water phantom. (b): SAD 100 cm, depth 11 cm in PMMA (12.6 cm water equivalent). ACPDP and D4 Interpolated reconstructions, and TPS calculation are all on a cylindrical homogeneous PMMA phantom.

**Figure 3:**
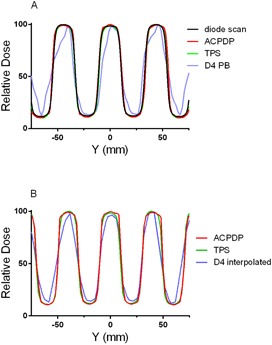
Relative lateral dose profiles in Y direction (in‐plane) for a bar pattern MLC‐defined field. (a): SSD 100 cm, depth 10 cm. The diode scan was taken in water, ACPDP and D4 PB reconstruction and TPS calculation are all on a Plastic Water phantom. (b): SAD 100 cm, depth 11 cm in PMMA. ACPDP and D4 Interpolated reconstructions, and TPS calculation are all on a cylindrical homogeneous PMMA phantom.

A set of cross‐plane (X) beam profiles in water or water‐equivalent material is presented in Fig. 2. As expected, a water phantom diode scan agrees well with the TPS calculation (Fig. 2(a)), since the MLC model optimization was based on a series of such scans. While ACPDP‐reconstructed profile exhibits reasonable agreement with the water scan, the D4 PB penumbra shape is substantially different. Figure 2(b) shows similar profiles reconstructed or calculated on a homogeneous PMMA phantom. While a diode scan in water is not available for such configuration, Fig. 2(a) demonstrates that the TPS profile can serve as a good approximation. A proven method of Delta^4^ volumetric dose reconstruction, D4 Interpolation, produces better agreement in the penumbra region with the TPS and ACPDP profiles. Figure 3 demonstrates a similar trend for the profiles taken in the in‐plane (Y) direction for a series of rectangular MLC openings (a bar pattern). D4 Interpolation again reproduces the true profile shape better than D4 PB. However, a careful comparison of the Delta^4^ profile shapes in Figs. 3(a) and 3(b) indicates some similarities, suggestive of insufficient resolution of both dose reconstruction methods in this experiment. By looking at Fig. 2(a) and Fig. 3(a), one would a priori expect substantial errors in D4 PB composite dose reconstructed for modulated beams comprised of small segments.

### B. VMAT dose reconstruction

#### B.1 PMMA Delta^4^ cylindrical phantom

The γ‐analysis passing rates are presented in Table 1. One can see good agreement between ACPDP, and both directly measured and reconstructed (interpolated) Delta^4^ doses. The average γ passing rate with a rather stringent 2% (local normalization)/2 mm criteria combination exceeds 90%.

**Table 1:** Gamma analysis passing rates (%) comparing Delta^4^ directly measured (Detectors Only) or D4 Interpolated (Volumetric) dose distributions on the PMMA cylindrical phantom with ACPDP and TPS (See Fig. 1(a)). The mean values for five VMAT cases are presented with standard deviations and ranges

	*Delta^4^ vs. ACPDP*		*Delta^4^ vs. TPS*	
*Comparison Region*	γ(3/3)≤1	γ(2/2)≤1	γ(3/3) ≤1	γ(2/2)≤1
Detectors Only	99.1±0.6 (98.5−100)	94.1±4.7 (87.0−100)	99.2±0.9 (98.1−100)	96.4±3.6 (90.8−100)
Volumetric	98.2±1.3 (97.0−100)	92.8±3.9 (89.5−99.2)	98.0±2.1 (95.2−100)	91.2±5.0 (83.4−95.7)

#### B.2 Water‐equivalent Delta^4^ cylindrical virtual phantom

The results for D4 PB reconstruction on a homogeneous cylindrical phantom are quite different from the D4 Interpolation method results on a similar dataset (first data line in Table 2). Comparison with both ACPDP and TPS shows the average agreement rate dropping by more than 10 percentage points for each comparison and threshold combination.

**Table 2:** Volumetric gamma analysis passing rates (%) comparing D4 PB with ACPDP and TPS on a water‐equivalent cylindrical phantom and actual patient CT datasets (See Figs. 1(b) and (c)). The mean values for five VMAT cases are presented with standard deviations and ranges

	*Delta^4^ vs. ACPDP*		*Delta^4^ vs. TPS*	
*Comparison Dataset*	γ(3/3)≤1	γ(2/2)≤1	γ(3/3)≤1	γ(2/2)≤1
Water Cylinder	88.6±6.8 (81.2−96.1)	72.4±8.4 (62.1−81.1)	88.5±7.8 (78.7−96.8)	73.8±10.9 (60.0−84.8)
Patient CT	81.2±8.6 (70.4−90.4)	64.6±8.4 (56.5−74.7)	80.8±8.5 (70.2−90.8)	63.6±8.0 (66.2−72.2)

#### B.3 Patient OT datasets

Comparisons between D4 PB and ACPDP/TPS on the patient datasets show further deterioration of agreement compared to the homogeneous water‐equivalent phantom (Table 2). The mean γ passing rates are about 81% and 64% for the 3%/3 mm and 2%/2 mm criteria combinations, respectively, indicating substantial disagreement. Absolute dose profiles presented for three cases in 4, 5, 6 further illustrate this progressive deterioration of dosimetric agreement. While D4 PB differs from D4 Interpolation primarily in the high gradient regions (penumbra) on the homogeneous phantom (compare 4, 5, 6), substantial disagreement with both ACPDP and TPS is observed in the relatively flat, high‐dose areas on the patients' CT datasets (4, 5, 6). This disagreement is reflected in the low gamma analysis passing rates (D4 PB versus ACPDP) noted in the figures for individual cases.

**Figure 4:**
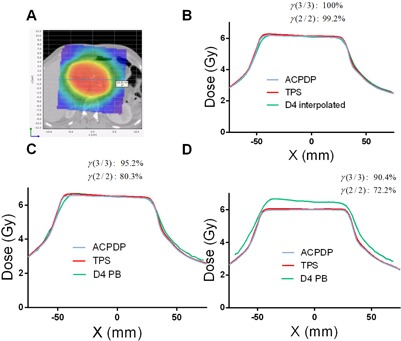
Absolute dose profiles for a Pancreas SBRT case. (a): Dose distribution and a Left‐Right (X) profile location relative to it. (b): Reconstruction/calculation on the Delta^4^ cylindrical PMMA phantom – D4 Interpolation vs. ACPDP vs. TPS. (c): Reconstruction/calculation on the water‐equivalent Delta^4^ phantom – D4 PB vs. ACPDP vs. TPS. (d): Same as for (c), but on the patient dataset. Gamma passing rates are presented comparing volumetric dose distributions from ACPDP and an appropriate D4 reconstruction method, on the corresponding phantoms.

**Figure 5:**
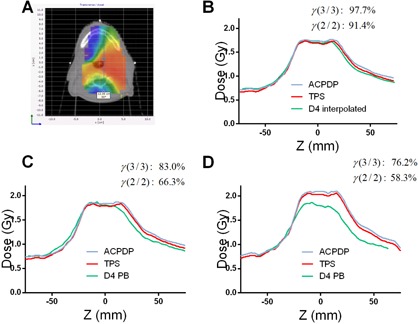
Absolute dose profiles for a head and neck case. (a): Dose distribution and an antero–posterior (Z) profile location relative to it. (b): Reconstruction/calculation on the Delta^4^ cylindrical PMMA phantom – D4 Interpolation vs. ACPDP vs. TPS. (c): Rreconstruction/calculation on the water‐equivalent Delta^4^ phantom – D4 PB vs. ACPDP vs. TPS. (d): Same as for (c), but on the patient dataset. Gamma passing rates are presented comparing volumetric dose distributions from ACPDP and an appropriate D4 reconstruction method, on the corresponding phantoms.

**Figure 6:**
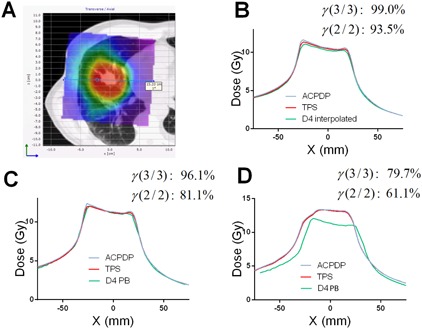
Absolute dose profiles for a Lung SBRT case. (a): Dose distribution and a Left‐Right (X) profile location relative to it. (b): Reconstruction/calculation on the Delta^4^ cylindrical PMMA phantom – D4 Interpolation vs. ACPDP vs. TPS. (c): Reconstruction/calculation on the water‐equivalent Delta^4^ phantom – D4 PB vs. ACPDP vs. TPS. (d): Same as for (c), but on the patient dataset. Gamma passing rates are presented comparing volumetric dose distributions from ACPDP and an appropriate D4 reconstruction method, on the corresponding phantoms.

For both 2%/2 mm and 3%/3 mm threshold combinations, matched analysis of variance (ANOVA) multiple comparisons showed statistically significant differences in mean agreement rates (p<0.05) for D4 Interpolation versus ACPDP on one hand, and D4 PB versus ACPDP on either cylindrical or patient dataset on the other hand. The differences were statistically insignificant for D4 PB versus ACPDP between the cylindrical phantom and patient datasets. The combined (pre multiple comparisons) repeated measures ANOVA test yielded highly significant p‐values for both the 2%/2 mm and criteria 3%/3 mm, 0.0001 and 0.003, respectively.

## IV. DISCUSSION

The D4 PB semiempirical dose reconstruction method was evaluated with a variety of dosimeters and methods. First, the dose profiles for small MLC‐defined static fields were examined against water phantom scans with a small detector. Volumetric comparisons were based on the measurement‐guided dose reconstruction with an independent dosimeter (ACPDP). This 3D dose reconstruction method is by now thoroughly validated, including direct, full 3D, gel dosimetry.^17^, ^25^, ^27^ It provided a satisfactory level of agreement at the 2%/2 mm level with both direct Delta^4^ measurements at the diodes' locations and D4 Interpolation (Table 1). Finally, while it is inappropriate to use TPS calculations as a sole benchmark for evaluating accuracy of a dosimetry system, a previously validated TPS provides additional confirmation.

All tests point towards the fact that the D4 PB algorithm produces substantial errors. It cannot accurately predict the penumbra shape for small MLC‐defined fields (2, 3), which is crucial for correct reconstruction of the modulated beams. At the same time, the D4 PB algorithm has no trouble accurately representing the umbra of a dose profile along the long dimension of an open $3\times 15\;{\text{cm}}^{2}$ field.^(22)^ Our profiles for the static fields are consistent with the graphs provided in the vendor's White Paper.^(22)^ D4 Interpolation, using exactly the same measurement data, generally produces better agreement. Reconstructing bar pattern profiles (Fig. 3), however, proved to be a challenging task for both interpolation algorithms. The shape of the D4 PB profile in Fig. 3(a) is consistent with insufficient spatial resolution, as expected with the energy fluence pixel size of $6\times 6\;{\text{mm}}^{2}$.^(22)^ Although the D4 Interpolation profile in Fig. 3(b) is in better agreement with the TPS, the residual errors with similar features are seen. The White Paper states: “Due to the highly irregular nature and limited resolution of the energy fluence matrix, deviations between calculated and measured dose in the Delta^4^ detector positions are more frequent in the IMRT case, … Deviations are primarily localized to regions of the rapid dose changes, indicating distances to agreement less than the pixel size, i. e. <6 mm.” It is clear that when the penumbra of individual segments is not represented with sufficient resolution, the superposition of many segments in a modulated beam would lead to rather inaccurate calculations. This is effectively equivalent to calculating modulated beams with a $6\times 6\;{\text{mm}}^{2}$ pixel size, while it is well established in the literature that segmented beam calculations require 2.5 mm voxel grid resolution to faithfully reproduce encountered gradients.^28^, ^29^, ^30^ This explains poor dosimetric agreement for VMAT plans found in Table 2 and 4, 5, 6. The profiles for a H&N case in the White Paper^(22)^ show errors similar in pattern and magnitude to our results.

No user‐adjustable parameters are available to fine‐tune the shape of the penumbra. While D4 PB shows fairly poor agreement with ACPDP and TPS when volumetric VMAT dose distributions are compared on a homogeneous cylindrical phantom, D4 Interpolation using the same exact measurement data produces much better agreement with both the ACPDP recon‐struction and TPS calculation (Table 1, 4, 5, 6). This demonstrates that intelligent interpolation techniques can largely overcome the relatively coarse spatial resolution of the measurement array,^16^, ^17^ but this appears not be the case in the implementation of the D4 PB algorithm. The White Paper points out that “the algorithm yields penambrae of the same width as the distance between measurement positions.”

Finally, the agreement between D4 PB and ACPDP/TPS is even worse when volumetric comparisons are performed on the patients' CT datasets (Table 2, 4, 5, 6). The difference with the homogeneous phantom did not rise to the level of statistical significance, likely due to the limited number of data points (five plans). The agreement tends to be worse in more heterogeneous media (head and neck and lung versus abdomen), suggesting possible additional issues with heterogeneity corrections. At this point, an attempt to provide further explanation of the D4 PB behavior in heterogeneous datasets is moot, given often poor agreement on a homogeneous phantom and the “black box” nature of the software, essentially with no user‐adjustable relevant parameters. Of course, substantial inaccuracies are inherently expected in the lung, particularly at the tumor/lung interface due to the well‐known limitations of the pencil beam dose calculation algorithm.^(31)^

## V. CONCLUSIONS

In summary, ACPDP, TPS and D4 Interpolation agree reasonably well at the 2%/2 mm level. On the other hand, D4 PB, based on the exact same Delta^4^ measurements as D4 Interpolation, shows poor agreement with ACPDP, TPS, and water scans. Heterogeneous CT datasets present the biggest challenge. Modern electronic dosimetry arrays are sophisticated systems comprised of hardware, firmware, and software. An additional level of complexity is added when the measured dose on relatively sparse detectors is used to reconstruct a high‐resolution volumetric dose grid throughout the phantom. The next step of dose reconstruction on the patient CT dataset can be even more complex. While this approach has the potential to provide for more intuitive and clinically useful evaluation of the patient‐specific, end‐to‐end tests, every system must be thoroughly tested before clinical use.

## ACKNOWLEDGMENTS

Moffitt Cancer Center has a Sponsored Research Agreement with Sun Nuclear Corp. However this work was not a part of that agreement and did not receive any external funding.
